# Issues determining direct airways hyperresponsiveness in mice

**DOI:** 10.3389/fphys.2012.00408

**Published:** 2012-10-22

**Authors:** Lennart K. A. Lundblad

**Affiliations:** Department of Medicine, Vermont Lung Center, The University of VermontBurlington, VT, USA

**Keywords:** Hyperresponsiveness, forced oscillation, unrestrained plethysmography, lung volume, mouse models of asthma

## Abstract

Airways hyperresponsiveness (AHR) is frequently a primary outcome in mouse models of asthma. There are, however, a number of variables that may affect the outcome of such measurements and the interpretation of the results. This article highlights issues that should be kept in mind when designing experiments using AHR as an outcome by reviewing techniques commonly used to assess AHR (unrestrained plethysmography and respiratory input impedance using forced oscillations), discussing the relationship between structure and function and, then exploring how the localization of AHR evolves over time, how the airway epithelium may affect the kinetics of methacholine induced AHR and finally how lung volume and positive end expiratory pressure (PEEP) can be used as tools assessing respiratory mechanics.

## Introduction

Animal models of complex diseases suffer from general issues compared with the true human disease since we do not know when a model is complex enough to adequately represent the human disease, nor do we always know if the individual parts of the model have their true correlates in the human disease. We denote some human diseases complex because we do not understand the mechanisms involved in generating the phenotype but to complicate matters further, this likely means that individuals that show seemingly similar phenotypes could have different underlying mechanisms leading to that phenotype. Asthma is one of those complex inflammatory diseases manifesting to different degrees of severity and yet also share a similar phenotype characterized by e.g., cough, acute bronchoconstriction, inflammatory infiltration of the airways, and airways hyperresponsiveness (AHR). AHR is a cardinal finding in asthma and animal models are almost invariably tested for AHR but unlike, say, determining the titer of a cytokine or counting cells in a lung lavage, AHR is measured by triggering a physiological response assessing the effects on lung mechanics. While this sounds straight forward there are multiple ways of challenging the test subjects and there are also different techniques used to measure lung mechanics all of which are not equally well equipped to generate high quality data. As a consequence not only the way AHR is assessed matters but also how AHR was induced in the first place ads to the perceived complexity. We can also ask what physical events in the airways determine AHR and how they develop over time. In this review I will try to point out some issues that can affect the results and the interpretation of respiratory mechanics measurements.

## Does the measurement technique matter?

### The forced oscillation technique

As alluded to in the Introduction the technique whereby AHR is measured does matter. While it can be claimed that some approaches are better than other, none is identical to the techniques used in humans. It is important to realize that our understanding of lung mechanics depends on our interpretation of a mathematical model of the respiratory system. The diagnosis of clinical asthma depends not only on physician's auscultation. It would be hard to imagine a doctor's diagnosis of asthma relying on auscultations only; objective assessments e.g., spirometry add to the diagnosis. In laboratory animals studies of physiology are based on mathematical models of the respiratory system, frequently using invasive techniques making the experiments terminal in nature. Unfortunately there are few alternatives to invasive techniques if quality data are to be obtained (Bates and Irvin, 2003). The complex respiratory system is often represented with a simplified model of an elastic compartment at the end of a resistive pipe. An analogy would be to think of a pipe with a balloon at the end where the dimension of the pipe contributes to the resistance (*R*) and the property of the balloon contributes to the elastance (*E*) of the system, although *R* and *E* have multiple sources throughout the respiratory system. At its simplest form calculating lung mechanics involves fitting an equation of motion to the variables (flow, volume, and pressure) measured during a ventilatory maneuver at a single frequency and then determining parameters of *E* and *R* (Equation 1).

(1) $$\Delta P=EV+R\dot{V}$$

However, due to the viscoelasticity of the respiratory tissues together with heterogeneities of the mechanical function of the lung values of *R* and *E*, obtained according to Equation 1, vary markedly with the frequency with which the respiratory system is oscillated, even over the range of normal breathing frequency (Similowski and Bates, 1991).

Another caveat is that the lung can demonstrate non-linear mechanical properties depending on the circumstances under which it is studied. E.g., when airflow in the airways become turbulent the *R*$\dot{V}$ term becomes non-linear or when the lung parenchyma is over distended the *EV* term becomes non-linear (Martin et al., 1988; Bates et al., 1997; Kaminsky et al., 1997; Wagers et al., 2002). Just determining *E* and *R* at a single frequency does not provide the investigator with a complete characterization of airway mechanics. Nevertheless, the simplicity of the model makes it attractive.

Another approach yielding more information about the respiratory system is to measure the impedance of the respiratory system (*Z*_rs_) at multiple frequencies. The respiratory impedance is a measure of how much the air and tissue of the respiratory system resists motion when exposed to a volume change over time. The value of *Z*_rs_ depends both on resistive as well as elastic forces of the respiratory system (the pipe and balloon, see above) and can vary greatly over frequency, hence, measuring *Z*_rs_ involves perturbing the respiratory system with a broad-band volume waveform containing many different frequencies simultaneously. Assuming that the respiratory system behaves in a linear way, this allows us to study the frequency response of the system. This gives us information on how the amplitude of a given input sine wave is scaled to produce its corresponding output sine wave of the same frequency. It also tells us how the phase of the input sine wave shifted. The input and output can be decomposed into their respective sine wave components by doing a Fourier transform. *Z*_rs_ is then determined for each individual frequency converting *P* and *V* in the time domain to their equivalent representation in the frequency domain. We can now examine the components at each frequency for the input and the output and see how their amplitude and phase differ. *Z*_rs_ is expressed in its equivalent representation by a complex number with a real and an imaginary part (Bates, 2009). At this stage *Z*_rs_ is only an empirical representation of the respiratory system despite containing all information about this system. We ultimately want to relate this information to structural elements of the respiratory system. This necessitates the use of mathematical models of the respiratory system. Hence, if there is a good match between the impedances of the model and the measurement, we may be able to say something about the actual condition of the measured lung. One such model that has gained popularity over the last couple of decades is the *constant phase model*, first introduced by Hantos et al. (Hantos et al., 1992),

(2) $$Z_{\text{rs}}(\omega )=R_{N}+i(\omega )I+\frac{G-iH}{{\left(\omega /\omega_{0}\right)}^{\alpha }}$$

where *R*_*N*_ is Newtonian resistance, *I* inertance, *G* tissue dampening and *H* elastance. *R*_*N*_ is the resistance of the conducting airways and primarily reflects the larger airways, hence, an increase in *R*_*N*_ implies narrowed airways. *I* depend on the mass of the air being oscillated; due to the small dimensions of mouse lungs this parameter can be ignored in mice but could be significant in larger species. *G* reflects loss of energy in the lung tissue and *H* is a measure of the stiffness of the lung. If *G* and *H* increase proportionally this can be interpreted as a loss of lung units where as if *G* increases more than *H* would indicate regional heterogeneities (Bates, 2009).

This approach has turned into today's gold standard of animal lung mechanics often referred to as the forced oscillation technique (FOT). The FOT was pioneered by (Dubois et al., 1956) but it was not until the arrival of powerful computers and the development of relevant mathematical lung models that the FOT became a commercialized standard technique in many lung laboratories (Schuessler and Bates, 1995). The advantage of using FOT in combination with the constant phase model is that it affords separation of the respiratory mechanics into effects on conducting airways (*R*_*N*_ and *I*) and effects on tissue (*G* and *H*) (Hantos et al., 1992; Bates, 2009).

### Unrestrained plethysmography

Unrestrained plethysmography has a surprisingly long history dating back to the late nineteenth century (Bert, 1868) but did not gain popularity until the advent of modern computers and the increased use of mice in pharmacological and physiological research. Its use was substantially boosted when the technique was commercialized in the 1990's. This was obviously fuelled by the demand from the bio-molecular and genetic research community for a quick and easy way to phenotype animals and to quantify effects of interventions without having to sacrifice the animal. Unrestrained plethysmography involves placing an animal inside a sealed chamber, within which it is allowed to move around freely, while the pressure changes induced by the respiratory movements are measured inside the chamber. Currently a quantity known as *Penh*, derived from the pressure swings inside the box, is used as a means to study respiratory mechanics in a range of animal models. The fundamental assumption behind *Penh* is that the breathing pattern produced by an animal in a closed chamber somehow correlates in a predictable way with airway resistance and that those changes correlate with airway physiology. *Penh* is a dimensionless quantity which affords a general characterization of the breathing pattern, but it is not based on a mathematical model of the respiratory system. *Penh* has been used in many studies as a measure of bronchial responsiveness (Chand et al., 1993; Hamelmann et al., 1997; Chong et al., 1998; Finotto et al., 2001; Lloyd et al., 2001).

Some studies have found a good correlation between *Penh* and other, more traditional, measures of airway mechanics (Hamelmann et al., 1997; Dohi et al., 1999; Finotto et al., 2001) but there is also evidence that *Penh* does not correlate with morphometric changes in mice (Lundblad et al., 1999). Further, it has also been shown that, under some circumstances, *Penh* may behave in an opposite way compared with a direct, invasive measurement of airway mechanics (Petak et al., 2001). In a recent ambitious study comparing *Penh* with FOT again results show that several genes implicated in AHR correlates differently depending on the phenotyping technique used (Berndt et al., 2011).

In a letter to the editor Mitzner and Tankersly (Mitzner and Tankersley, 1998) raised the issue, from a theoretical point of view, that the pressures generated by a breathing animal inside the unrestrained plethysmograph will arise from two sources: (1) gas compression and (2) gas conditioning. We later showed that gas compression and gas de-compression of thoracic gas occurs with changes in intrathoracic pressure, which drives the expiratory and inspiratory flow. Gas conditioning, on the other hand, occurs when the air moves from inside the box into the lungs of the animal. The air in the box is relatively cool and dry and when it enters the animal's lungs it gets humidified and heated to body temperature. This leads to an expansion of the gas inside the animal and, it is impossible to separate between the two sources contributing to box pressure merely from the changes in box pressure (Lundblad et al., 2002). Yet another problem with unrestrained plethysmography is that it appears to be strain specific; results from two of the most commonly used mouse strains, Balb/C and C57Bl6 tend to be different and it is believed that *Penh* largely originate as part of reflex control of breathing processes, rather than in the mechanics of the lung (Adler et al., 2004). Needless to say there has been a continued debate over the validity of unrestrained plethysmography (Mitzner et al., 2003; Bates et al., 2004; Sly et al., 2005; Lundblad et al., 2007a; Hantos et al., 2011a,b). If unrestrained plethysmography were to generate valid estimates of respiratory mechanics we need more information than just fluctuations of plethysmographic pressure; we need to know the lung volume. Indeed we expanded the unrestrained plethysmography concept by deriving a new quantity that reflects respiratory mechanics rather than just breathing pattern. This new quantity was denoted IPPI (Inspiratory Plethysmographic Pressure Integral) (Lundblad et al., 2002). Since the publication of this study we constructed a video-assisted plethysmograph where the changes in lung volume were determined via orthogonal video imaging of the thorax. These estimates were combined with the pressure swings recorded as mice breathe inside a heated and humidified plethysmograph yielding an estimate of specific airway resistance (Bates et al., 2008b).

While it might be tempting to use *Penh* as a crude first attempt trying to determine if e.g., a treatment is effective on AHR there are problems arising at several levels with this approach. First, *Penh* is not based on first principles but is a mere measure of breathing pattern using ratios of swings in plethysmograph pressure vs. inhalation and exhalation times to calculate an index that does not have anything to do with airways resistance or lung stiffness. Second, the signal generated by an unrestrained plethysmograph depends almost entirely on conditioning of the temperature and humidity of the inhaled air. Indeed, only when the airways are severely constricted would unrestrained plethysmography generate data related to airways resistance. Third, mice are obligatory nose breathers and there is, of course, no way to separate effects on the nose and the lung without an invasive measurement bypassing the nose similarly to what has been done in guinea pigs (Finney and Forsberg, 1994).

While we safely can discard the use of unrestrained plethysmography, for reasons described above, techniques based on first principles of the physics of the respiratory system, such as the models discussed above (Equations 1 and 2), have merit because it is possible to understand these techniques based on the mathematical model of the respiratory system they are based on, albeit with varying degrees of precision and resolution, and we can design experiments to determine the validity of the models. Do we need to control everything while generating high quality data but sacrificing natural animal behavior or can we accept less data precision but avoiding anesthesia and paralysis? This conundrum was coined “The Phenotyping Uncertainty Principal” (Bates and Irvin, 2003). Eventually, the choice of method always comes down to the question one is trying to answer.

## The localization of AHR

AHR might be present in different parts of the lung. While traditionally thought of as an impairment of airflow localized to the conducting airways we now know from imaging studies in asthmatic humans that the physiological consequences of asthma manifests throughout the lung in its entirety. It has been shown that the ventilatory pattern of the asthmatic human lung is impaired compared with healthy lungs in that the asthmatic lung tends to have heterogenous ventilation patterns. Imaging in the form of positron emission tomography (PET) was used to show that interdependence among the airways in the asthmatic lung can lead to the emergence of self-organizing patterns of airway narrowing and closure ultimately leading to ventilation defects. As a result, even the presence of minimal heterogeneity in the airway narrowing can lead to large clusters of lung being poorly ventilated during an asthma attack and that even for uniform smooth muscle activation of a symmetric bronchial tree; the presence of minimal heterogeneity breaks the symmetry and leads to large clusters of poorly ventilated lung units (Venegas et al., 2005; Harris et al., 2006; Winkler and Venegas, 2011). Their findings suggest a mechanism of topographical distribution of self-organizing ventilation defects that is reminiscent of other emergent processes throughout biology and yet the phenomenon is not random. In a recent study from the Woolcock Institute using Single Photon Emission Computed Tomography (SPECT) it was shown in asthmatics exposed to inhaled methacholine that an increase in non-ventilated lung volume correlated with peripheral conductive abnormality and with AHR (Farrow et al., 2012). Those observations yet again support the notion that AHR is dominated by peripheral events in the lung such as airway closure and heterogeneous ventilation. It is important to note that despite the study subjects being supine airway closure appears preferentially in the lower lung regions, suggesting that the underlying disease rather than posture is the underlying cause of ventilation defects.

It might not come as a big surprise that humans with a long history of inflammatory lung disease would have disturbed ventilatory patterns of their lungs. The findings in human asthmatics raise the question whether animal models of asthma share a similar phenotype in terms of ventilation irregularities. Using a computational model of the mouse lung, and comparing with *in vivo* impedance data from mice it was shown that the AHR in allergically inflamed Balb/C mice was distinctly different from that of spontaneously hyperresponsive mice (A/J) or Balb/C mice treated intratracheally with polycations disrupting the airway epithelium. The AHR of allergic mice were characterized by increased lung stiffness whereas A/J and polycation treated Balb/C mice had an AHR dominated by increased resistance of the conducting airways probably due to smooth muscle contraction (Bates et al., 2006; Wagers et al., 2007). In studies using computer modeling of the respiratory system and comparing lung mechanics data, from the constant phase model, with micro-CT imaging we discovered that allergic mice, with a much shorter disease history than human patients, also show patterns of airway closure. This was accomplished by ventilating mice with 100% oxygen before methacholine challenge. When airway closure occurred after methacholine exposure oxygen trapped below the closures was absorbed by the circulation and the resulting atelectatic lung regions were then visualized and quantified by micro-CT. Indeed our data strongly supported the notion that AHR in the allergic mouse depends almost entirely on peripheral airway closure and that the volume of lung lost to atelectasis accounted exactly for the increase in lung elastance. Moreover, computer modeling show that thickening of the airway wall combined with an increased propensity for airways to close explain the loss of functional lung during an episode of AHR, while the properties of the airway smooth muscle remains normal (Wagers et al., 2004; Lundblad et al., 2007b). In support of our previous studies it was recently found, using hyperpolarized ^3^He magnetic resonance imaging, that the ventilatory pattern of the lungs in allergically inflamed mice was significantly impaired even before being exposed to methacholine with the periphery of the lung being most affected (Thomas et al., 2012).

Most studies of AHR tend to focus on a time point where the response is at a maximum, e.g., the peak of airways resistance. Interestingly the localization of AHR is not static over time. We recently found that the geographical location of AHR changes dramatically over several weeks. Mice were sensitized to ovalbumin (OVA) with two i.p. injections separated by 14 days and then AHR was measured 16 h post a single OVA inhalation on day 21 (*Short Challenge* group), after 3 days of OVA inhalation on day 25 (*Standard Challenge* group) and following an OVA inhalation on day 55 in mice previously challenged on days 21–23 (*Recall Challenge* group). The results show that AHR in the *Short Challenge* group was characterized by an increase in conducting airway resistance and neutrophil accumulation in the lavage whereas AHR in the *Standard Challenge* group was characterized by increases in lung elastance and tissue dampening but by only a modest response in resistance, while inflammation was eosinophilic. AHR in the *Recall Challenge* group was characterized by increases only in elastance and tissue dampening and elevated numbers of both neutrophils and eosinophils. Hence, AHR can vary both in nature and severity over time and does so concomitant with variations in inflammation showing that AHR is an evolving phenotype. This raises the possibility that we might be better served studying this variability rather than simply relying on a single time point where AHR is maximal (Riesenfeld et al., 2012).

Imaging techniques have developed considerably over the past decades and whereas advanced imaging techniques used to be reserved for human use they are now available for laboratory use. While historically used primarily to elucidate structural changes of tissue, imaging techniques can also be used to elucidate alteration of e.g., gas volume and gas distribution in the lung. While most imaging studies have been done in humans some interesting findings have also been made in laboratory animals, although very few in mice. As discussed above, imaging techniques have been used in human as well as in animal studies and indeed appear to show that similar if not identical phenomena are present in our animal models as in the human disease in terms of ventilation irregularities and distribution of patchy airway closure. The use of imaging techniques has thus provided us with important insight into the pathophysiology of asthma and allowed us to draw important conclusions about structure and function. Imaging techniques come with their own set of issues in terms of how to correctly perform scans of various types, use of contrast etc. Easily recognizable issues from a physiological point of view are the same as for performing high quality lung mechanics measurements; lung volume and positive end expiratory pressure (PEEP) must be closely monitored and standardized. Absorption atelectasis (oxygen absorption by blood circulation of oxygen trapped in alveoli) is another issue to keep in mind since the air contains 21% oxygen there is a real potential that lung volumes can vary considerably. We typically ventilate mice with inert nitrogen before scanning to avoid this phenomenon. In our studies using micro-CT the animals were dead and scanned at *rigor mortis* because even the slightest movement of the animal would have affected the scan and the subsequent analysis of structural elements of the lung and gas volume (Lundblad et al., 2005, 2007b; Schwartz et al., 2011).

## Does the challenge route matter?

AHR is only one component in the process of diagnosing of asthma and in the human setting a number of different agents and challenges have been and are being used to trigger the desired airway response. Examples are methacholine, adenosine mono-phosphate, mannitol, histamine, antigen, and cold air (Wesseling and Wouters, 1992; Nassenstein et al., 2008; van den Berge et al., 2010; Suh et al., 2011; Anderson and Lipworth, 2012). In animal models the by far dominating agent used is methacholine and very few studies have used anything else making true comparisons with human studies a bit of a challenge. Methacholine is a parasympathomimetic with a longer half-life than the endogenous nervous transmitter acetylcholine and is a full agonist on the muscarinic receptor (Markovic et al., 2012). It is eliminated via the same routes as acetylcholine via breakdown by cholinesterases and removal by blood circulation. Thus, while methacholine is readily dissolvable in physiological solutions and diffuses easily across epithelial and endothelial barriers it is still vulnerable to elimination before reaching the receptors on the smooth muscle. Thus, it is conceivable that the rate by which methacholine diffuses across biological barriers could have an impact on the receptor mediated response from purely kinetic reasons. Indeed, using a mechanically based computer model, we recently showed that the increased responsiveness of the airway smooth muscle can, to a large part, be explained by decreased diffusion barrier of the airway epithelial lining (Cojocaru et al., 2008; Bates et al., 2012). Interestingly it has also been shown that administering methacholine intravenously in allergic mouse models of asthma generates a response dominated by smooth muscle contractions in the conducting airways whereas the response produced by inhaled methacholine is dominated by peripheral airway closure (Lundblad et al., 2007b; Bates et al., 2009). This discrepancy between inhaled and intravenous administration of methacholine raises a number of important questions pertaining to why and how this happens but perhaps more importantly how the interpretation of data is affected.

## Diffusion across epithelial barrier

AHR is typically assessed as a response to a chemical agent such as inhaled methacholine. It is assumed that the response generated in the lung by the smooth muscle is related to the inhaled methacholine dose and that the administered dose correctly corresponds to the dose reaching the smooth muscle. This is probably not true, however, since it is well known that the deposition of aerosol particles is significantly influenced by the inhalation pattern, flow, aerosol droplet size and also the geometry of the airway tree (Ma and Darquenne, 2011; Tian et al., 2011). The effect of using different nebulizers on airway deposition in humans was elegantly demonstrated using radioactive labelled aerosol of technetium-99 m-labeled diethylene triamine pentaacetic acid [(99 m)Tc-DTPA] where the deposition pattern varied from central airways to peripheral lung with decreasing particle size (Bondesson et al., 2007). In rabbits it was shown that rapid-shallow breathing combined with larger aerosol droplets tended to increase deposition in the central airways whereas deep-slow ventilation rates favored deposition in the lung periphery (Dahlback et al., 1994). In yet another human study it was found that the fall in specific airway conductance was significantly larger after deposition in the main bronchi than in the peripheral airways (Schmekel et al., 1994), illustrating that the distribution of agonist can significantly affect the airway response and subsequently the interpretation of the results. Furthermore, the dose delivered (lung burden) will vary with the degree of bronchoconstriction and it has been suggested that the response to incremental doses of inhaled methacholine is self-limiting because severely constricted airways prevents further deposition of agonist (Brown and Mitzner, 1998). The picture is further complicated by the fact that the inhaled agonist need to travel across several barriers of mucus, epithelial cells, connective tissue and interstitium before reaching the smooth muscle during which time the compound is subjected to elimination by enzymatic breakdown and removal by the circulation (Kelly et al., 1986a,b; Wagner and Mitzner, 1990, 1995). This situation is not constant but varies with the inflammatory state of the airway tissue. We have previously shown that the time to peak response in mice inhaling methacholine increased with inflammation (Wagers et al., 2004, 2007) whereas it decreased when the epithelial barrier function was disrupted by poly-L-lysine (Bates et al., 2006, 2008a). We were recently able to present a model showing quantitatively how the kinetics of methacholine transport and elimination affect the AHR in mice (Bates et al., 2012). We found that not only do the barriers contribute significantly to the time-course of bronchoconstriction but the results suggest that alterations in the diffusion pattern across these barriers might play an important role in diseases with significant remodeling such as inflammatory airways diseases. It is known that methacholine has significant effects on the pulmonary circulation (Robertson et al., 2010) likely accounting for the hypoxemia after intra venous methacholine administration. Inhaled methacholine, on the other hand, induces extensive airway closure, as reviewed above, however it was recently shown in sheep that inhaled methacholine did not effectively affect the smooth muscle of the bronchovascular circulation (McLeod et al., 2012). It is not clear if this is also the case in the mouse; although it could be speculated that the extent of airway derecruitment seen in mice might cause transient systemic effects e.g. hypoxia.

At any rate, the examples above illustrate that AHR can manifest in geographically different locations depending on the route of the challenge, deposition pattern, regional kinetics and direct effects on circulation.

## Lung volume and ventilation

The volume history of a lung will affect the lung mechanics. This becomes very apparent when comparing healthy vs. asthmatic subjects where a deep inhalation protects the healthy lung against bronchoconstriction whereas the protective effect is abolished in asthmatics (Fish et al., 1981). While the lung volume is typically not controlled in human test subjects during pulmonary function tests, the situation is the opposite in anesthetized and tracheotomized animals where a PEEP is commonly applied. Of course, PEEP is not equal to lung volume because the resulting volume of a set pressure could conceivably vary with the state of the lung, e.g., an inflamed, stiff lung would have a lower lung volume compared with a healthy lung (Lundblad et al., 2007b) and an emphysematous lung would have a larger volume than normal (Lundblad et al., 2005). Nevertheless, PEEP offers a backdrop of constant airway pressure against which lung mechanics is measured and the level of PEEP can have significant effects on the impedance of the lung because of the effects on lung volume. In a study by Sly et al. it was shown that when PEEP = 0 cmH_2_O *R*_*N*_ was at its highest but decreased progressively with increasing PEEP. At the same time *G* and *H* were at their lowest at PEEP = 0 cmH_2_O and increased with increasing PEEP (Sly et al., 2003). This study illustrated the importance of keeping the lung volume and/or PEEP under strict control since even a relatively small change in PEEP might add to the experimental error and potentially obliterate differences between study groups or erroneously imply differences.

It is also possible to use lung volume as a tool to elucidate the condition of a lung. Remember, not all inflammatory conditions render the lung hyperresponsive and thus a methacholine challenge might not always be useful. However, by determining the impedance at different lung volumes or PEEP it is possible to assess if an injured lung has elevated elastance at a lower lung volume than a control lung. This would be indicative of a consolidating process or fibrotic process in the lung tissue. We did a comprehensive analysis of the phenotypic consequences of TNFα overexpression in the lung. The lungs of these mice were severely injured and we found no difference in respiratory mechanics between TNFα overexpressing mice and controls at normal PEEP = 3 cmH_2_O, however, when PEEP was increased to 6 cmH_2_O we found that the injured mice had stiffer lungs as evidenced by elevated elastance. Micro computed tomography scans and histology confirmed that TNFα had caused significant consolidations and fibrotic lesions of the lungs (Lundblad et al., 2005). We were, thus, able to link structure to function by manipulating PEEP, measuring impedance and doing advanced imaging of the lungs.

In this context it is important to point out that high PEEP or deep insufflations can also affect the smooth muscle response in significant ways (Shinozuka et al., 1998; Hirai et al., 1999; Hirai and Bates, 2001). To study if deep inhalations offer a bronchodilatory effect we performed a study in normal Balb/C mice. The mice received an intra-venous methacholine bolus and then we measured the evolving bronchoconstriction for 16 s. During this time we delivered 1 ml deep inhalations at various time points. The results show that the development of bronchoconstriction is PEEP dependent such that lower PEEP generates higher *R*_*N*_ than higher PEEP. When a deep inhalation was delivered the bronchoconstriction was reduced but continued to increase although not reaching the same level as in mice not receiving a deep inhalation (Bates et al., 2007). We speculate that the explanation for the post deep inhalation reduction in responsiveness could be due to a reduction in the forces of circumferential interdependence between the airway smooth muscle and the airway wall caused by sudden stretching of viscoelastic attachments. At any rate, a deep inflation had significant effects on the development of airways resistance again illustrating how lung volume changes can modulate an induced response and affect the interpretation of an experiment. In this context it is important to distinguish between protective effects afforded by a deep inspiration that prevents airways from constricting, from that of a dilatory effect generated by a deep inspiration. The latter would reduce the degree of e.g., methacholine induced bronchoconstriction and reinstate some level of airway patency. When we delivered a deep inhalation post i.v. methacholine we observed a drop in resistance, indicative of a bronchodilatation. In another experiment of our study the deep inhalation was delivered 8–9 s after an i.v. bolus of methacholine and then a new dose of methacholine was delivered at 5 s later. Contrary to the predictions of our computational airway model the respiratory resistance did not reach the same level as in control mice not receiving any deep inhalations. This points to the possibility of a bronchoprotective effect and not only a bronchodilatory effect (Bates et al., 2007). The set up to test animals for AHR typically involves anesthesia, tracheostomy and extra corporeal ventilation. This situation is obviously far from the conditions under which the same animal would breathe were it to be conscious. As it turns out the ventilation pattern applied to the animal can have significant effects on the AHR measurement. The bronchoconstriction elicited by methacholine is highly sensitive to both the ventilation frequency and the tidal volume. In a study in rabbits it was found that either increasing the tidal volume or increasing ventilation frequency significantly suppressed the methacholine induced bronchoconstriction (Shen et al., 1997). Similar results were later shown in mice where the minute volume was kept constant. Again the bronchoconstriction was antagonized when the tidal volume was increased, however, it was also discovered that A/J mice were more sensitive to changes in tidal volume than C57Bl/6 mice (Chen et al., 2006). Although the nature and origin of AHR may not be fully understood the smooth muscle is involved in some fashion. Using the data from (Shen et al., 1997) and applying a two dimensional model of an airway tethered to the parenchyma Bates and Lauzon (Bates and Lauzon, 2007) showed that a large part of the dynamics of bronchoconstriction can be understood in terms of the relative stiffness of the parenchyma and airways, despite the tissue being exposed to cyclically shortening and lengthening. It appears that the shape of force velocity curve of the tissue is preserved but that the increase in ventilation amplitude keeps pulling the tissue not allowing full contraction of the muscle.

We can conclude that PEEP and lung volume as well as lung volume history and ventilation pattern all have significant effects on the parameters of respiratory mechanics and as a consequence must be closely monitored and kept constant throughout a study lest results from different animals renders the investigation useless.

## Summary

The techniques available to phenotype the respiratory system in animal models have developed significantly and so has our understanding of the possibilities and limitations of the methods available to the researcher. Assessing AHR in models of asthma will continue to be important as an endpoint for drug effects but also in determining the relevance of potential drug targets and understanding pathways involved in the pathology of inflammatory lung diseases. In this overview I have attempted to highlight some aspects of AHR assessment that can influence the interpretation of the animal model. The choice of measurement technique and mathematical model can have dramatic effects on the interpretation of the result and the validity of the conclusions from a study. Likewise, the administration route of the agonist, e.g., methacholine, and the kinetics by which the agonist migrates over tissue boundaries will significantly affect the outcome of the AHR measurement. The way an experiment is designed with respect to standardization of ventilation pattern and lung volume history can also affect the outcome dramatically.

### Conflict of interest statement

The author declares that the research was conducted in the absence of any commercial or financial relationships that could be construed as a potential conflict of interest.
